# The Potential of Personalized Virtual Reality in Palliative Care: A Feasibility Trial

**DOI:** 10.1177/1049909121994299

**Published:** 2021-02-15

**Authors:** Letizia Perna, MSc, MSW, Sam Lund, Nicola White, Ollie Minton

**Affiliations:** 1 9098 Royal Trinity Hospice, London, UK; 2 Marie Curie Palliative Care Research Department, 4919 UCL, London, UK; 3 4968 St George’s University Hospitals NHS Foundation Trust, London, UK; 4 Brighton & Sussex NHS Trust, Brighton, UK.

**Keywords:** hospices, virtual reality, symptom assessment, palliative care

## Abstract

**Background:**

Virtual Reality can help alleviate symptoms in a non-palliative care population. Personalized therapy can further alleviate these symptoms. There is little evidence in a palliative care population.

**Aim:**

To understand the feasibility of repeated personalized virtual reality sessions in a palliative care population.

**Design:**

A feasibility randomized control trial. Intervention: personalized virtual reality, Control: non-personalized virtual reality. All participants completed a 4-minute virtual reality session for 4 weeks. At each point, the Edmonton Symptom Assessment System-Revised (scored 0 = none up to 100 = worst) was completed pre- and post- each session. A time-series regression analysis was completed for the overall effect.

**Setting/Participants:**

The research took place in one hospice. The main inclusion criteria was: (1) under the care of the hospice (2) advanced disease (3) over 18 years (4) physically able to use virtual reality set (5) capacity (6) proficient English.

**Results:**

Twenty-six participants enrolled, of which 20 (77%) completed all sessions. At baseline, the intervention group had a mean pre- score of 26.3 (SD 15.1) which reduced to 11.5 (SD 12.6) after the first session. At the same time point, the control group had a mean pre- score of 37.9 (SD 21.6) which reduced to 25.5 (SD 17.4) post-session. The mean scores dropped following each session, however this was not significant (mean difference = −1.3, 95% CI: −6.4 to 3.7, p = 0.601).

**Conclusions:**

It is feasible to complete repeated virtual reality sessions within a palliative care population. Future research should explore the structure and effectiveness of virtual reality in a fully powered trial.

## Background

Virtual reality (VR) is a technology which generates computerized 360-degree images and sounds that give the viewer a sense of physical presence in that environment through the use of a headset/goggles and headphones. A previous study investigating the impact of VR for people who have experienced a stroke showed an improvement using the Edmonton Symptom Assessment System (ESAS).^1^ Since its development, VR therapy using computer generated environments have shown to have positive effects in pain management,^2-4^ Post-Traumatic Stress Disorder (PTSD),^5^ anxiety,^6^ and depression.^7^ These symptoms are commonly reported within a palliative care population,^8^ and therefore it is possible that VR could provide a non-pharmacological alternative to alleviate them, alongside the holistic model of care that hospices in the UK follow.^9,10^

Evidence for VR in a palliative care setting is sparse, however there have been 2 international studies that have done this.^11,12^ Both studies used a single-arm design and used a single VR session (lasting 30 minutes). Both studies found trends for improvements on symptoms following VR using the Edmonton Symptom Assessment System-Revised (ESAS-R). Johnson et al^12^ reported that participants in their study felt that multiple sessions would be more useful than a single session. Neither of these studies explored the possibility of personalizing the content of the VR. Research using still images with a healthy population concluded that personally emotive images trigger stronger positive physiological and psychological responses.^13^ Therefore it is possible that a personalized VR session, could elicit a stronger response. To the authors’ knowledge, there have been no UK studies investigating multiple personalized VR sessions in a palliative care population.

The aims of this study were to test (1) the feasibility and acceptability of recruiting people with advanced illness in to a trial with multiple VR Sessions; and (2) whether outcomes on the ESAS show any effect of personalized VR.

## Methods

This report follows CONSORT reporting guidelines for pilot and feasibility trials^14^ as well as the Template for Intervention Description and Replication (TIDieR) checklist^15^ to describe the intervention.

### Trial Design

A single site feasibility non-blind randomized control trial

### Participants

Inclusion criteria: (1) Under the care of the hospice (2) Progressive life limiting condition(s) (3) Aged 18 years or over (4) Full visual and auditory abilities (5) No restriction in their range of head and neck movement (6) Capacity to consent to the study (7) Fluent in English.

Exclusion criteria: a diagnosis of dementia in the medical notes.

Enrolment was either self-enrolment (through posters and leaflets placed in clinical and non-clinical areas of the hospice) or caseload review by a hospice staff member. There was no dedicated researcher for this study; instead, the hospice clinical team (doctors, nurses, and allied health professionals) were aware of the study and identified eligible participants as part of their routine clinical work. The hospice team made the initial approach to the participant and, if they agreed, the author (LP) discussed the study in more detail. If the patient self-enrolled to the study, a member of the hospice team would check their eligibility before contacting the researcher. If the participant wished to continue, a structured capacity assessment following the Mental Capacity Act^16^ was completed (by the researcher) and consent was obtained for those with capacity. Recruitment was completed between October 2017 and June 2019. The hospice is located in the south west of London with inpatient and community services. It has 28 beds over 2 floors and last year, the hospice served over 2,500 people.

The target sample size was 26, based on pragmatic grounds of demonstrating feasibility, acceptance, and likely attrition.

### Intervention

After providing written consent, participants were randomized by the researcher in to 1 of 2 groups: personalized (intervention) or non-personalized (control) using an online platform (www.randomizer.org). The researcher and participant were not blinded to this allocation.

If the patient was able to come to the hospice, the sessions were carried out in a private outpatient clinic room. If the patient was home-bound, then the sessions happened in the patient’s home. If the patient was admitted to our ward, the session happened in their hospice room. There was no time limit for each session, to accommodate the participant and their potential fluctuating health needs.

**Figure 1.**
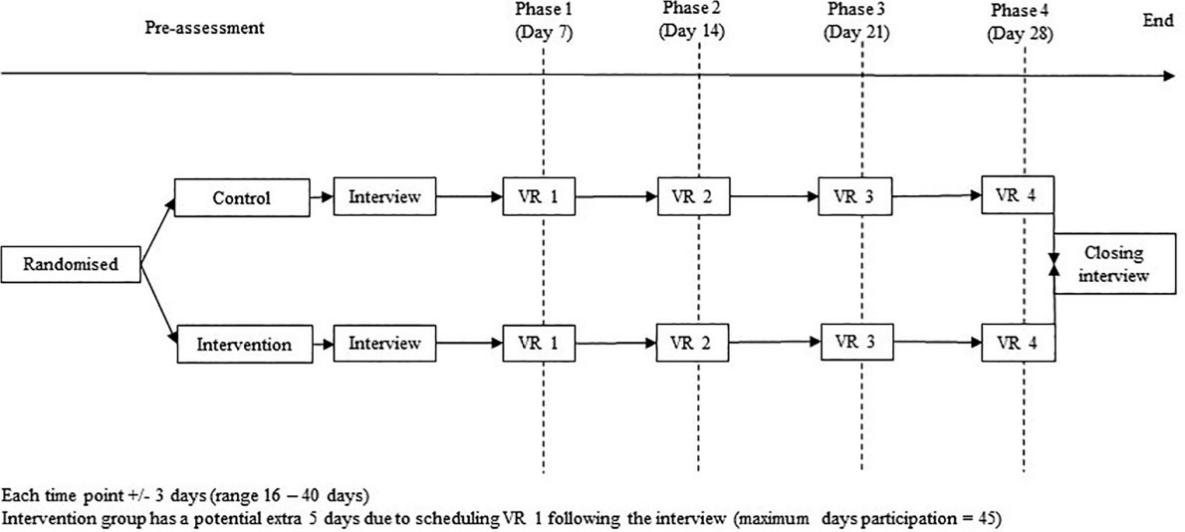
Study flowchart.

See Figure 1 for the study flowchart. All participants completed four 4-minute VR sessions, once a week (± 3 days to account for flexibility with an inherently unstable population) with the researcher and Flix Films. All sessions took place in the hospice. The length of the high quality videos online determined the length of the VR session. The number of sessions was based on the hospice’s standard model of therapy (8 sessions), which was halved in order to reduce participation burden.

VR was delivered through a Google Daydream headset using a Google Pixel XL smartphone and headphones. The content was sourced and made possible for use in a clinical setting by Flix Films from publicly available video libraries such as YouTube. See Supplementary File 1 for a list of video content.

#### Personalized group (Intervention)

Participants randomized to the intervention group completed a personalized content interview, which took up to 30 minutes to complete, (see Supplementary File 2 for Interview topic guides for each arm) with the researcher to obtain preferences for the VR session (such as their favorite opera). However, this was then limited to what was publically available on the internet. The interview occurred within 5 working days of obtaining consent.

#### Non-personalized group (Control)

Participants randomized to the control group were asked about any potential phobias/fears. The 4 VR sessions for this group were randomly selected from a set of 6 pre-selected experiences, excluding any experience that might be related to a fear/phobia identified during the interview.

The content of the experiences was determined by the available content sources by Flix Films and the study author (LP) due to their length and generic content.

Once consent had been given and the interview completed, the participant and researcher scheduled the first VR session (a date within 5 working days of completing the interview). During each session, the date for the following session was organized.

### Outcomes

#### Feasibility of recruitment and retention

- The recruitment target was 26 people.
- Rates and reason for attrition.
- Rates and reason for any sessions being stopped before the VR ended.

#### Acceptability of VR sessions

- As done in a previous feasibility trial,^17^ acceptability was determined by the attrition data. Acceptable feasibility criterion whereby 60% of participants engage at least partly with the VR sessions according to this criteria:
  - No acceptability (0/4 sessions)
  - Part acceptability (1-3/4 sessions)
  - Full acceptability (4/4 sessions)

#### Quantitative measures

The Edmonton Symptom Assessment System-Revised (ESAS-R) is a validated tool with 10 domains.^18-21^ Each domain is scored ranging from 0 (none, best) to 10 (worst). The ESAS-R was completed by participants pre- and post- each VR session.

### Statistical Methods

Following an Intention-to-treat analysis, all data was included and reported.

The demographic data (age, sex, diagnosis) of the participants are described.

A total score (maximum = 100) for the ESAS-R was calculated. The mean and standard deviation (SD) ESAS-R scores were generated by participant and by session. A linear regression analysis was completed to compare the mean ESAS-R between the study arms at each session. Each session was adjusted for the baseline score.

The mean difference between the pre- and post-ESAS-R scores was calculated at each session and compared between arms. A time-series regression analysis was completed for the overall effect of the VR experience between the trial arms over the sessions.

The mean difference between the pre- and post-ESAS-R scores for each domain were calculated. This was not statistically analyzed due to the low sample size, but the results are described for full transparency.

STATA v14.0 was used for all analyses.

Ethical approval was received from St Georges University of London (SGREC17.0017).

## Results

**Figure 2.**
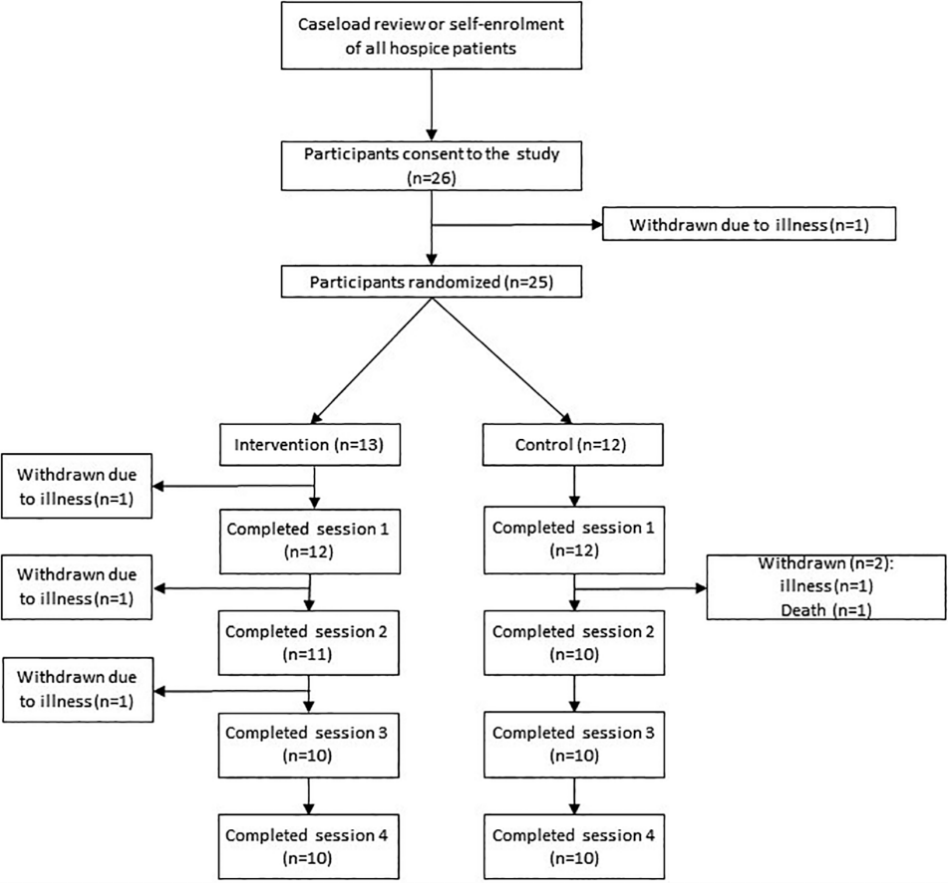
Recruitment flowchart.

There were 26 participants were randomized into the study, of which 20 completed all sessions (Figure 2). Drop out was due to clinical deterioration and death (n = 6). Participants who started each session were able to complete the full 4 minutes.

There were 14 women (70%) and 6 men (30%) with a mean age of 66 (27-85 range). There were 15 participants (75%) with cancer and 5 (25%) with other life limiting diseases. Study participation lasted between 16 and 45 days.

### Feasibility of Recruitment

It took 20 months to identify and recruit 26 patients who were eligible for the study. Everyone who the researcher approached to participate in the study agreed to do so. This suggests it is feasible to recruit patients with advanced illness to a VR trial. The main barrier to recruitment was due to not having an assigned researcher to conduct the research. The researcher (LP) completing the trial was also a full time staff member within the hospice.

### Acceptability of VR Sessions

No participant asked for the VR session to be stopped during the session. As shown in Figure 2, 20/25 (80%) of all participants who were randomized [10/13 (77%) intervention; 10/12 (83%) control; 1 not randomized due to illness], met the criteria for “Full acceptability.” There was 1/25 (4%) participant who met the criteria for “No acceptability,” and 4 (16%) participants who met the criteria for “Partial acceptability.” There was one further participant who consented but not randomized due to illness. These results show that the acceptability criterion of 60% has been surpassed, indicating that multiple VR sessions are acceptable to individuals with advanced disease in a hospice setting.

The overall ESAS-R score pre VR was 29.8 (SD 19.2; range 1-79), and post-VR session was 19.9 (SD 18.7; range 0-76).

### Quantitative Measure

Table 1 describes the mean ESAS-R scores by phase and by group.

**Table 1.** Mean ESAS Scores (Total/100) by Group and Session.

	Total	Intervention	Control
	Mean (SD)		
**Session 1**			
Participants (n)	24	12	12
Baseline	32.1 (19.1)	26.3 (15.1)	37.9 (21.6)
Post-VR	18.5 (16.5)	11.5 (12.6)	25.5 (17.4)
**Session 2**			
Participants (n)	21	11	10
Baseline	28.5 (18.3)	24.8 (16.2)	32.7 (20.3)
Post-VR	17.7 (14.4)	13.0 (11.3)	22.8 (16.1)
**Session 3**			
Participants (n)	20	10	10
Baseline	31.8 (20.1)	22.9 (11.2)	40.7 (23.4)
Post-VR	24.7 (21.4)	16.5 (15.5)	32.8 (24.1)
**Session 4**			
Participants (n)	20	10	10
Baseline	26.2 (20.2)	18.9 (11.0)	33.5 (24.9)
Post-VR	19.3 (22.6)	10.3 (9.6)	28.2 (28.4)

There was no overall statistical difference in the mean difference in pre- and post-ESAS-R scores overall between those who received the intervention and those in the control group (mean difference = −1.3, 95% CI: −6.4 to 3.7, p = 0.601) (Table 2).

**Table 2.** Mean Difference Between Pre and Post VR ESAS Total Scores.

	Intervention	Control	Intervention	Control	Mean difference	95% CI	*P* value	Adj. p value
Session	*n*		*Mean (SD)*					
**1**	12	12	−14.8 (9.1)	−12.4 (9.7)	−2.4	−10.4 to 5.6	0.536	-
**2**	11	10	−11.8 (8.8)	−9.9 (9.4)	−2.0	−10.3 to 6.4	0.626	0.971
**3**	10	10	−6.4 (17.3)	−7.9 (7.5)	1.5	−11.0 to 14.1	0.805	0.823
**4**	10	10	−8.6 (6.7)	−5.3 (8.7)	−3.3	−10.5 to 4.0	0.354	0.545

Table 3 displays the mean difference between the pre- and post-ESAS-R scores by domain. Those who completed the “others” domain, listed issues such as circulation, headaches, itchiness, bowel problems, tremors, hot flushes, incontinence, and neuropathic pain. Those in the intervention group appeared to experience the biggest reduction in tiredness, anxiety, and psychological wellbeing scores. The control group seemed to experience the biggest reduction in tiredness and drowsiness scores.

**Table 3.** ESAS-R Domains by Phase.

	Session 1	Session 2	Session 3	Session 4
	Mean difference (SD)			
**Intervention**				
**N**	12	11	10	10
Pain	−2.2 (2.8)	−1.4 (1.6)	−1.6 (2.0)	−1.0 (1.2)
Tiredness	−2.6 (2.2)	−1.9 (1.8)	−1.7 (2.3)	−1.4 (1.5)
Drowsiness	−1.4 (1.9)	−1.4 (1.9)	−1.0 (2.1)	−1.5 (2.5)
Nausea	−0.3 (0.6)	−0.2 (0.6)	0.5 (3.5)	−0.1 (0.3)
Appetite	−0.3 (0.5)	−1.3 (1.6)	0.1 (2.7)	−1.0 (1.7)
SOB	−1.2 (2.4)	−0.3 (0.7)	−0.8 (1.6)	−0.7 (1.1)*
Depression	−1.8 (1.7)	−1.4 (1.7)	−0.7 (1.6)	−0.2 (0.4)
Anxiety	−2.2 (1.7)	−1.7 (1.9)	−0.8 (3.0)	−1.4 (2.1)
+ Wellbeing	−2.9 (2.2)	−2.3 (1.8)	−1.1 (2.7)	−1.4 (1.3)
Other	−1.3 (1.5) n = 3	−0.5 (0.7) n = 2	3.5 (6.4) n = 2	0.0 (0.0) n = 2
**Control**				
** N**	12	10	10	10
Pain	−0.7 (1.6)	−1.0 (1.9)	−1.1 (1.6)	−0.9 (1.7)
Tiredness	−2.5 (2.2)	−1.1 (1.3)	−1.9 (2.6)	−1.5 (1.5)
Drowsiness	−1.7 (2.1)	−1.5 (2.2)	−1.1 (1.3)	−0.3 (0.9)
Nausea	−0.8 (1.6)	−0.2 (0.6)	−0.1 (0.6)	0.3 (0.7)
Appetite	−0.7 (1.5)	−0.4 (0.7)	0.0 (0.0)	−0.1 (0.3)
SOB	−0.8 (1.3)	−1.3 (1.7)	−0.6 (1.1)	−0.1 (1.5)
Depression	−0.9 (1.9)	−1.2 (1.6)	−0.2 (0.8)	0.0 (0.5)
Anxiety	−1.4 (1.4)	−1.4 (1.6)	−1.9 (2.6)	−1.8 (3.3)
+ Wellbeing	−1.8 (1.4)	−1.4 (1.9)	−0.7 (1.2)	−1.0 (1.7)
Other	−2.3 (2.6) n = 6	−0.7 (1.0) n = 6	0.5 (0.8) n = 6	0.2 (0.4) n = 2

*1 missing (Phase 4, n = 9); “+ wellbeing”: psychological wellbeing.

## Discussion

### Main Findings

It is feasible and acceptable to recruit people with advanced illness in a hospice setting into a trial with multiple sessions of VR. Of the participants who completed the first session, 20/25 (83%) completed all 4 sessions.

Repeated VR (regardless of which group the participant was assigned to) did seem to improve some of the psychological and physical symptoms, but this difference was not statistically significant. Personalized VR did not significantly improve ESAS-R scores over non-personalized VR.

### Strengths and Limitations

This is a novel study in the UK investigating personalized VR, over repeated sessions, for people with a terminal illness.

However, limitations to this study design might caveat this research finding and warrant further research. This was a small sample, single site with a skewed methodological sample collection and there was no blinding to the VR interventions. The VR sessions in this research were not as long as previous studies (4 minutes compared to 30 minutes) and it could be possible that a longer session at each time might have elicited a different response. There is also a limitation with the VR content for the study arms. The personalized content was restricted to what was available on the online public library and the control group inadvertently included some videos that were personalized for the participants (e.g. it was a video of a “bucket list” destination). While every effort was made to limit this, it might have lessened any potential effects between the groups. These limitations reduce the generalizability of the findings. The lack of funding for a dedicated researcher to the trial affected the recruitment phase and the length of the trial. No data were stored of the number of records screened to reach the sample size, as recruitment was dependent on referrals from the clinical staff in the hospice as part of their routine work. In order to appraise the feasibility of VR fully within this population, we recommend a dedicated researcher and a screening log as part of a larger funded trial.

### What This Study Adds

This research suggests it is feasible to recruit people with advanced illness, in a hospice setting, for a repeated VR trial. While preliminary findings suggest a reduction in ESAS-R scores, there were no statistically significant findings. This is comparative to the previous work,^11,12^ and provides the data to inform a future larger trial.

Future studies, in a fully powered trial, should explore the structure of VR (session length and number of sessions) to further understand the clinical benefit to patients under palliative care services.

## Supplemental Material

Supplemental Material, sj-pdf-1-ajh-10.1177_1049909121994299 - The Potential of Personalized Virtual Reality in Palliative Care: A Feasibility Trial Supplemental Material, sj-pdf-1-ajh-10.1177_1049909121994299 for The Potential of Personalized Virtual Reality in Palliative Care: A Feasibility Trial by Letizia Perna, MSc, MSW, Sam Lund, Nicola White and Ollie Minton in American Journal of Hospice and Palliative Medicine®

Supplemental Material, sj-pdf-2-ajh-10.1177_1049909121994299 - The Potential of Personalized Virtual Reality in Palliative Care: A Feasibility Trial Supplemental Material, sj-pdf-2-ajh-10.1177_1049909121994299 for The Potential of Personalized Virtual Reality in Palliative Care: A Feasibility Trial by Letizia Perna, MSc, MSW, Sam Lund, Nicola White and Ollie Minton in American Journal of Hospice and Palliative Medicine®
