# Supplementary material for: The Potential of Personalized Virtual Reality in Palliative Care: A Feasibility Trial
Source: Am J Hosp Palliat Care. 2021 Feb 15;38(12):1488–94. doi: 10.1177/1049909121994299 (PMC8641032; doi:10.1177/1049909121994299)
Supplement: Supplemental Material, sj-pdf-1-ajh-10.1177_1049909121994299 - The Potential of Personalized Virtual Reality in Palliative Care: A Feasibility Trial [file sj-pdf-1-ajh-10.1177_1049909121994299.pdf]

Supplementary File 1 VR content in the arms

Control	Intervention
Swimming with dolphins	African safari
Hot air balloon ride	Angel falls
New York	Ayers Rock/Ululu
Northern Lights	Disney
Sunrise	Swimming with dolphins
Venice	Egypt
	Elephant & hippos from a boat
	Encounters with wild animals
	Flying a plane
	Hang-gliding
	India
	Maldives
	New York
	New Zealand
	Northern lights
	Opera
	Paris
	Rome
	Sky diving
	Sunrise
	Venice
	Music festival
