# Supplementary material for: The Potential of Personalized Virtual Reality in Palliative Care: A Feasibility Trial
Source: Am J Hosp Palliat Care. 2021 Feb 15;38(12):1488–94. doi: 10.1177/1049909121994299 (PMC8641032; doi:10.1177/1049909121994299)
Supplement: Supplemental Material, sj-pdf-2-ajh-10.1177_1049909121994299 - The Potential of Personalized Virtual Reality in Palliative Care: A Feasibility Trial [file sj-pdf-2-ajh-10.1177_1049909121994299.pdf]

Interview Topic Guides

Personalised Content Arm

At the start

Member of the research team introduces self to the participant.

Member of the research team explains to the participant that the interview will be audio recorded.

Explanation of scope of interview to participant: You have been randomly selected to experience virtual reality experiences which will be personalised to your tastes, wishes, hobbies and bucket list. In order for us to create your personalised virtual reality experiences throughout this study, we would like to ask you a few questions to understand what experiences or locations connect with you most from an emotive point of view and make you feel happiest.

Prompts will be used to guide the interview.

Standardised questions

- Please list up to 4 things you wish you could have done or places you wish you could have seen that are on your bucket list.
- Please list up to 4 of your most memorable experiences or places you have visited
- Please tell us a little bit about your interests and hobbies.
- Please tell us about what you most miss since being diagnosed.
- Please tell us what gives you joy.
- What activities or events in your life have made you feel happiest?
- Is there anything you've wanted to try in life / place you've wanted to visit, and weren't able?
- Are there any sounds / music / tones that you find most soothing?
- Would you describe yourself as a sporty or laidback person?
- Are you prone to motion sickness or dizziness?
- Have you any phobias, i.e. Heights, falling, water, enclosed spaces, insects/animals?
- Did you grow up in a Rural or Urban environment?
- Do you prefer watching the ocean or walking through a forest?
- Is there a particular animal that gives you comfort?
- What is the one experience you would most like to have through this project?

Conclusion

Thank you for your time and for answering our questions. As mentioned your answers will help us create your personalised virtual reality experiences which you will be experiencing over the course of the study. At this time we would like to schedule a time for your participation in the first phase of the study.

Non-Personalised Content Arm

At the start

Member of the research team introduces self to the participant.

Member of the research team explains to the participant that the interview will be audio recorded.

Explanation of scope of interview to participant: You have been randomly selected to experience virtual reality experiences which will be pre-selected for you by our research team. We want to make sure that your virtual reality experiences do not cause you unnecessary distress through exposure to any of your phobias. With that in mind, we wanted to discuss what your phobias are.

Prompts will be used to guide the interview.

Standardised questions

- Are you prone to motion sickness or dizziness?
- Have you any phobias, i.e. Heights, falling, water, enclosed spaces, insects/animals?

Conclusion

Thank you for your time and for answering our questions. As mentioned your answers will help us create your virtual reality experiences which you will be experiencing over the course of the study. At this time we would like to schedule a time for your participation in the first phase of the study.
